# Supplementary material for: NKG2D Fine-Tunes the Local Inflammatory Response in Colorectal Cancer
Source: Cancers (Basel). 2023 Mar 16;15(6):1792. doi: 10.3390/cancers15061792 (PMC10046042; doi:10.3390/cancers15061792)
Supplement: Supplementary file 1 [file cancers-15-01792-s001.zip › cancers-2228589-supplementary.pdf]

cancers MDPI

Supplementary Materials

Supplementary Table S1. Antibodies used in this study.

Antigen	Conjugate	Clone	Source
B220	PE-Cy7	RA3-6B2	BioLegend
CD11b	APC-eFluor780	M1/70	eBioscience
CD11b	PE-CF594	M1/70	BD
CD11b	PerCp-Cy5.5	M1/70	eBioscience
CD19	APC-eFluor780	1D3	eBioscience
CD19	BV650	6D5	BioLegend
CD27	BV510	LG.3A10	BD
CD27	PE/Dazzle594	LG.3A10	BioLegend
CD3	BV650	17A2	BioLegend
CD3	V450	17A2	BD
CD3	PE-CF594	145-2C11	BD
CD3	BUV737	145-2C11	BD
CD4	APC-eFluor780	GK1.5	Invitrogen
CD4	BV605	GK1.5	BioLegend
CD4	APC-eFluor780	RM4-5	eBioscience
CD4	BV711	RM4-5	BioLegend
CD4	BUV495	GK1.5	BD
CD44	PerCP-Cy5.5	IM7	BioLegend
CD45	BV605	30-F11	BioLegend
CD45	AF700	30-F11	BioLegend
CD8α	BUV395	53-6.7	BD
CD8α	APC-eFluor780	53-6.7	Invitrogen
CD8α	PerCp-Cy5.5	53-6.7	BioLegend
CD8α	BV785	53-6.7	BioLegend
CD8α	BUV395	53-6.7	BD
EpCAM	APC-eFluor780	G8.8	eBioscience
Fixable Aqua Dead Cell Stain Kit	N/A	N/A	ThermoFisher
Granzyme B	AlexaFluor-647	GB11	BioLegend
IFN-g	PE-Cy7	XMG1.2	eBioscience

IFN-γ	PE	XMG1.2	eBioscience
IL-17A	FITC	GL3	eBioscience
IL-17A	PE-Cy7	TC11-18H10.1	BioLegend
IL-17A	PE	eBio17B7	eBioscience
NK1.1	AlexaFluor-488	PK136	BioLegend
NK1.1	PE-Cy7	PK136	BioLegend
NKG2D	PE/Dazzle594	CX5	BioLegend
NKG2D	APC	CX5	BioLegend
NKG2D	PE	CX5	eBioscience
NKp46	BV421	29A1.4	BioLegend
PD-1	BV650	J43	BD
TCR Vg1	PE	2.11	BioLegend
TCR Vg4	APC	UC3-10A6	BioLegend
TCR Vg4	PeCy7	UC3-10A6	eBioscience
Zombie NIR	N/A	N/A	BioLegend
γδTCR	PerCp-eFluor 710	GL3	eBioscience
γδTCR	BV421	GL3	BD
γδTCR	APC	GL3	eBioscience
γδTCR	PE-Cy5	GL3	eBioscience
γδTCR	FITC	GL3	eBioscience

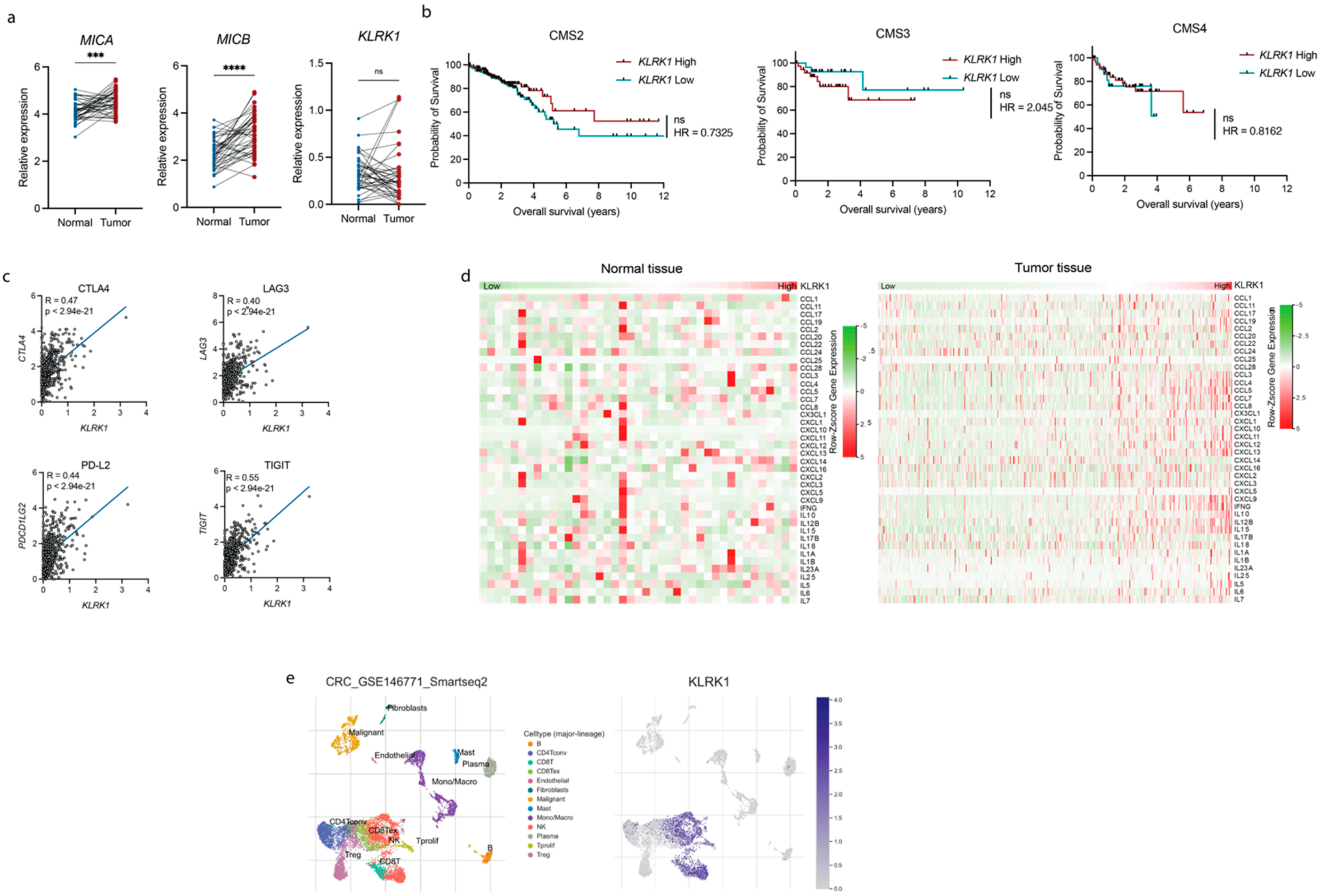

Supplementary Figure S1. (**a**) Relative expression of the NKG2D ligands *MICA* and *MICB* as well as *KLRK1* in tumor compared to adjacent healthy tissue of COAD patients. (**b**) Survival probability in patients expressing high (blue) vs. low (yellow) levels of *KLRK1* in different subtypes of colorectal cancer. (**c**) Correlation between markers of immune cell exhaustion and *KLRK1.* (**d**) Heatmap of genes associated with an inflammatory response in normal tissue (left) compared to tumor tissue of CRC patients (right). Each column represents one sample and samples are ordered from low to high *KLRK1* expression. (**e**) UMAP plots showing the major cell subsets and expression patterns of *KLRK1* in CRC patients. Statistical significance was determined using paired t-test or Wilcoxon matched-pairs signed rank test following Shapiro-Wilk normality test (a), Log-rank (Mantel-Cox) and Mantel-Haezenszel test comparing upper and lower percentile (b) and linear regression analysis (c). * $p \leq 0.05$, ** $p \leq 0.01$, *** $p \leq 0.001$, **** $p \leq 0.0001$.

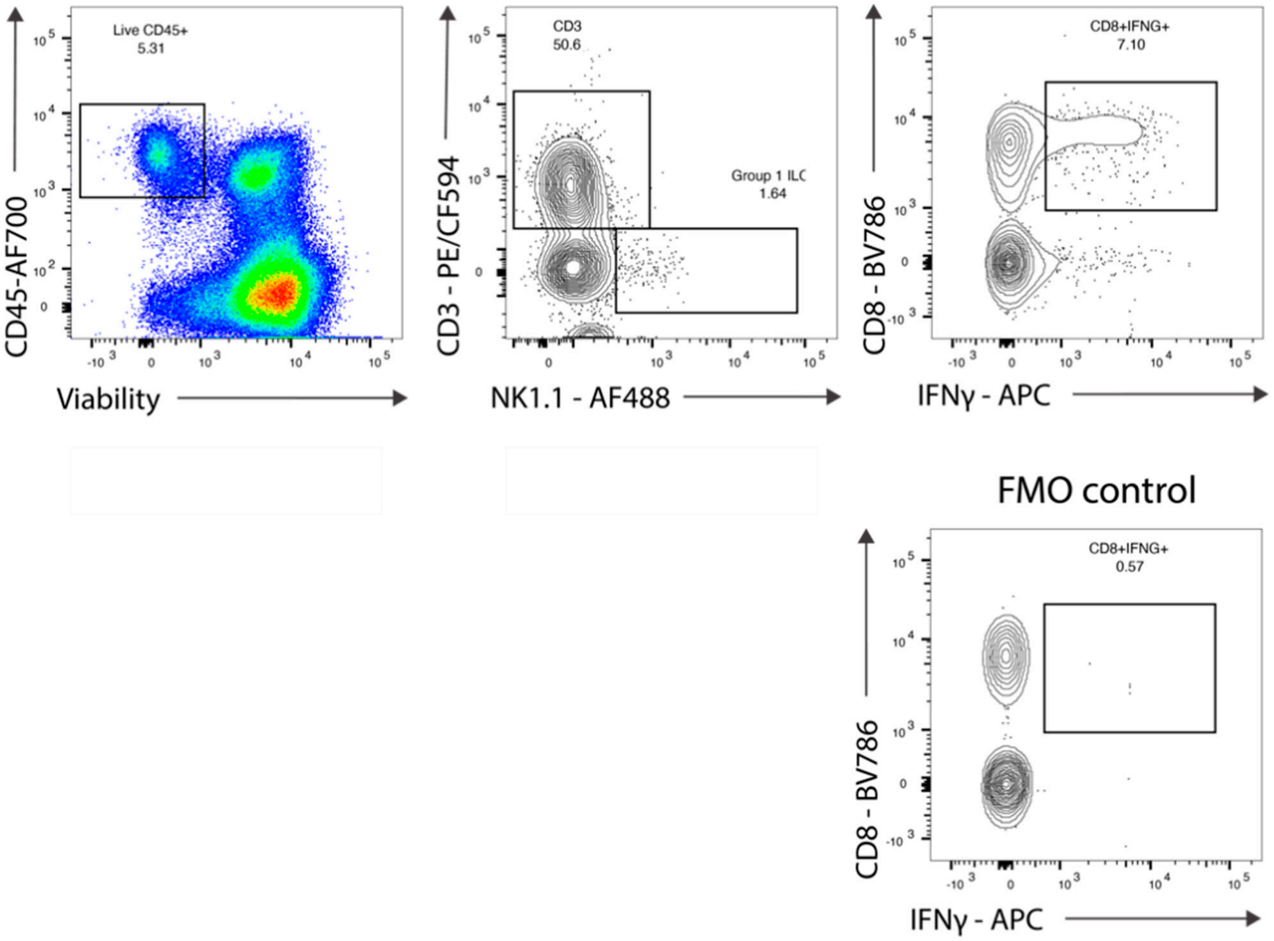

Supplementary Figure S2. Representative gating strategy to measure cytokine production by tumor-infiltrating CD8⁺ T cells. FMO = fluorescent minus one.
